# Caregiver Burden in Partners of Parkinsonian Patients with Deep Brain Stimulation

**DOI:** 10.3390/brainsci12020238

**Published:** 2022-02-09

**Authors:** Eileen Gülke, Monika Pötter-Nerger

**Affiliations:** Department of Neurology, University Medical Center Hamburg-Eppendorf, 20246 Hamburg, Germany; e.guelke@uke.de

**Keywords:** caregiver burden, Parkinson’s disease, deep brain stimulation, neuropsychiatric symptoms, depression

## Abstract

In Parkinson’s disease (PD) patients, the progressive nature of the disease and the variability of disabling motor and non-motor symptoms contribute to the growing caregiver burden of PD partners and conflicts in their relationships. Deep brain stimulation (DBS) improves PD symptoms and patients’ quality of life but necessitates an intensified therapy optimization after DBS surgery. This review illuminates caregiver burden in the context of DBS, framing both pre- and postoperative aspects. We aim to provide an overview of perioperative factors influencing caregiver burden and wish to stimulate further recognition of caregiver burden of PD patients with DBS.

## 1. Introduction

Caregiver burden (CB) is defined as “the extent to which caregivers perceive that caregiving has an adverse effect on their emotional, social, financial, physical and spiritual functioning” [1] and occurs in the context of providing informal care for relatives with chronic diseases. Since Parkinson’s disease (PD) is a complex disorder with increasing disabling motor and non-motor symptoms over time, partners of PD patients are at risk of increased CB. The interplay of motor disabilities such as bradykinesia or tremor and non-motor impairments such as cognitive decline, depression or urinary dysfunction challenges both caregiver and recipient. Along with disease progression, higher CB occurs in advanced stages of the disease with higher symptom severity [2], with non-motor symptoms impacting CB more than motor impairments [3]. General risk factors for CB are female sex, cohabilitation with the care recipient, the amount of caregiving time and effort and lack of choice [4]. Female caregivers of PD patients have worse quality of life (QOL) along with impaired mobility, emotional well-being and non-motor symptoms of the PD patient as predictors of CB [5]. CB can have detrimental effects on the quality of caregiving, as well as the mental health of the caregiver. Therefore, it is pivotal to engage further family members to uncover and reduce CB [4] and prevent premature institutionalization, as this does often not meet the wishes of caregiver and recipient [6].

In advanced disease stages, oral drugs fail to sufficiently control PD symptoms, with motor and non-motor fluctuations leaving the patient and caregiver with uncertainty and helplessness in part due to the loss of control of unpredictable symptoms. Therefore, device-aided therapies such as deep brain stimulation (DBS) represent a therapeutic option to provide a substantial long-term improvement of fluctuating PD symptoms, QOL [7] and probably longer life expectancy [8]. The DBS operation defines a turning point in the long course of the disease and comes along with high hopes and fears of the patient and caregiver. However, the difficult postoperative adjustments of medication and stimulation can result in severe side effects such as accentuated neuropsychiatric symptoms, potentially resulting in higher risk of suicide [9], which could affect postoperative CB. Little is known whether informal caregivers actually profit from DBS in terms of CB reduction along with postoperative motor and non-motor symptom control.

**Figure 1:**
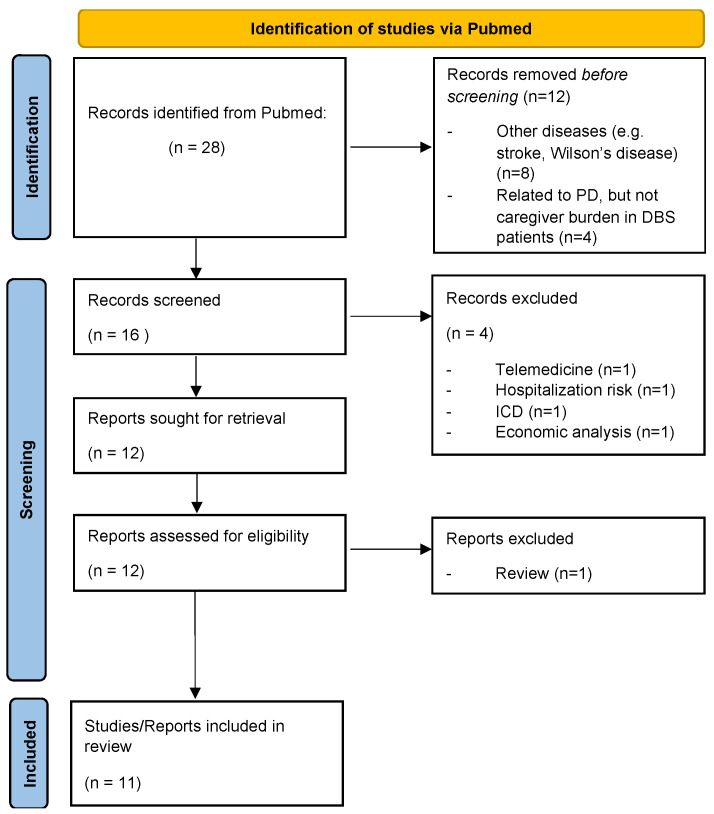
Flow diagram for search of PubMed database with the search terms “Caregiver Burden” and “Deep Brain Stimulation” or “DBS”.

This review aims to provide a concise overview of factors contributing to CB in PD in the context of DBS. We retrieved relevant literature published in the PubMed database from 1 January 1993, to 2 February 2022. Database searches were limited to articles published in English. The search terms were as follows: “Caregiver Burden” and “Deep Brain Stimulation” or “DBS”. (Figure 1) (Table 1). Additionally, studies of reference were also manually retrieved with the following search terms: “Caregiver Burden” or “Caregiver” or “Caregiving” and “Parkinson” or “Parkinson’s disease” or “Neuropsychiatric symptoms”. Titles and abstracts were screened, and more details were retrieved from the main manuscript. The following information was extracted from the included studies: first author, year of publication, study design, patient’s age, sample size, PD duration, gender distribution, number of caregivers enrolled and outcome parameters/domain of interests.

We first discuss preoperative aspects of PD influencing CB. Here, we concentrate on preoperative mediators of CB and then focus on care recipient and caregiver expectations of DBS. Second, we address postoperative issues arising from the DBS-induced symptom relief or DBS side effects and how that impacts postoperative CB. We discuss how DBS interferes with the long-built relationship of care recipient and caregiver when suddenly the burden of disease fades. Our target is to raise attention to this significant clinical problem and mitigate postoperative CB of PD patients’ caregivers.

## 2. Preoperative Caregiver Burden—DBS Yes or No?

### 2.1. Caregiver Burden Due to Insufficient PD Symptom Control in Advanced Stages

Optimal control of both motor and non-motor symptoms becomes difficult as the therapeutic window narrows in advanced stages of the disease. The combination of oral antiparkinsonian drugs results in a highly complex medication intake with tight timetables but often insufficient symptom control. Along with disease progression, caregiver demands increase, with the highest estimated cost for PD patients in Hoehn and Yahr stage IV [2]. Motor symptoms become more severe with occurrence of unpredictable motor fluctuations such as peak-dose dyskinesia, wearing off, gait dysfunction, freezing of gait, postural instability and higher risk of falls. Accordingly, caregiver burden is positively correlated with PD motor symptoms, overall disability and Hoehn and Yahr stage, as well as a decrease in PD patients’ activities in daily living (ADL) [2,10]. In fact, motor symptoms are a relevant factor for the prediction of CB in nonsurgical PD patients [11]. Among the diverse non-motor aspects of PD, neuropsychiatric symptoms such as depression, psychosis or impulse control disorders play a substantial role in the daily living of PD patients. These neuropsychiatric symptoms even contribute more to the caregiver burden of PD partners than motor impairment [2,5,12]. Caregiver distress increases along with worsening neuropsychiatric symptoms and lower quality of life of PD patients [13]. In a cross-sectional observational study of 89 PD patients and 84 caregivers, neuropsychiatric symptoms, such as mood changes and apathy but also MDS-Unified Parkinson’s Disease Rating Scale (MDS-UPDRS) II, caregiving hours per day and caregivers’ Beck Depression Inventory (BDI), had the most impact on CB [14]. Apart from that, cognitive disturbances correlate with PD patients’ quality of life and caregiver burden [11]. There are even extreme examples where PD patients cannot tolerate being alone at all [2]. Neuropsychiatric complications can result in physical aggression against the caregiver, also being directly connected with CB in advanced PD patients [15]. The 25-item self-reported Alzheimer’s Patient Partners Life Impact Questionnaire (APPLIQue) identified in 74 informal caregivers of advanced-stage PD patients in Hoehn and Yahr stage IV–V the three most rated aspects: “feel of guilt if not there” (71% affirmed), “situation wears me down” (65%) and “always on my mind” (61%) [16]. Factors associated with higher caregiver QOL were female PD patient gender, better PD patients’ cognition, lower non-motor symptom burden of PD patients and not being the partner of the patient [16]. In conclusion, PD challenges informal caregivers due to the multitude of different symptoms and caregiver responsibilities, with neuropsychiatric symptoms exerting the probably most severe impact on CB.

### 2.2. Caregiver Expectations of DBS

When oral antiparkinsonian drugs fail to sufficiently control motor symptoms, advanced device-aided therapies such as DBS serve as rescue options. Subthalamic nucleus deep brain stimulation (STN-DBS) is a well-established therapy for PD and now considered even earlier in the course of the disease when the first clinical signs of motor fluctuations and medically refractory symptoms such as tremor appear [17,18,19]. DBS treatment is associated with substantial symptomatic relief and maintenance of activities of daily living (ADL) even over 10 years [7]. The lower the preoperative quality of life (QOL), the higher the improvement in QOL after 24 months [20]. The combined effect of STN-DBS and L-dopa reduces motor severity in PD even more than either treatment alone [21], with a reduction in L-dopa equivalent following surgery of 55.9%, a 69.1% reduction in dyskinesia and an average reduction of off-periods of 68.2% [22]. Apart from motor improvement, non-motor symptom reduction was associated with QOL and ADL in a 24-month follow-up [23]. This is of great importance, as caregiver burden is highly dependent on non-motor symptomatology [24].

Therefore, DBS is often considered a “game changer” for both PD patients and their caregivers and raises high expectations of DBS effects on QOL. As to the great involvement of spouses in caregiving, the decision to undergo DBS surgery should take into account the caregiver’s expectations and fears. DBS represents an invasive operation of the brain with potential intraoperative complications such as intracranial bleeding, infection and the need for electrode revision [25], which might elicit fears and concerns in terms of intraoperative adverse events in patients and caregivers. Complication rates are low but must be disclosed to the caregiver and care recipient. It is discussed that unrealistic, unmet expectations contribute to postoperative caregiver dissatisfaction and increased CB [26]. Despite caregiving responsibilities, the PD subject and caregiver have an overall satisfying relationship preoperatively, but this relationship will be challenged by the sudden DBS-induced changes of motor capabilities of the care-receiving PD patient [27]. Both patients’ and caregivers’ treatment expectations should be preoperatively addressed and the risk–benefit ratio and perioperative demands of the procedure communicated [28]. A study from a single tertiary care hospital in India revealed a profound lack of knowledge and misconceptions regarding DBS among PD patients and caregivers [29]. Thereby, false expectations and postoperative disappointment can occur. In fact, a computer application (DBS-Edmonton app) improved DBS-related knowledge and patient satisfaction [30]. It is also important to discuss with patients and caregivers that regular postoperative follow-up visits are mandatory for DBS programming, hardware evaluation or battery checks and changes in medication [31]. In a large, multicenter cohort of movement disorder patients, 12.3% of caregivers recharged the impulse generator of the patient due to caregiver integration in the postoperative process that the caregiver should be aware of preoperatively [32]. Apart from that, PD patients’ as well as caregivers’ fear the idea of “becoming another person”, so the concept of an individual identity and possible, mostly transient, disease- and medication-related mood changes should be discussed prior to surgery [26,33]. In fact, PD patients’ and caregivers’ awareness of possible mood changes after DBS is still limited [26]. Nevertheless, a perioperative caregiver survey showed that two-thirds of caregivers feel fully prepared for DBS surgery but suggests more information on postoperative care and more communication with the family during surgery [34]. Importantly, a better understanding of the disease in PD caregivers is associated with lower CB [3], which supports the effort of intensified preoperative education of PD patients and caregivers. A perioperative psychoeducation program can reduce the anxiety of both PD patients and caregivers up to 2 years after surgery and can help to avoid postoperative maladjustment [35]. In summary, PD patients’ and caregivers’ expectations of DBS effects are high and must be adjusted by preoperative information and counseling.

## 3. Postoperative Aspects of Caregiver Burden

### 3.1. Caregiver Burden Due to PD Neuropsychiatric Symptoms after STN-DBS

Since PD neuropsychiatric symptoms represent generally one of the most challenging symptoms of CB, neuropsychiatric postoperative changes are highlighted here. Dopaminergic medication and STN-DBS act on the motor, limbic and associative basal ganglia circuit [36]. However, the corresponding segments of the subthalamic nucleus have their own dose–response curves with partly opposing pathological symptoms [36]. Postoperative titration of stimulation and medication primarily centers around optimal motor symptom control. On the one hand, neuropsychiatric symptoms such as apathy, anxiety and depression reflect hypodopaminergic states of the limbic and associative loops as a result of withdrawal of dopaminergic drugs. On the other hand, hyperdopaminergic symptoms such as mania or impulse control disorders may occur when stimulation spreads either in other basal ganglia circuits or surrounding anatomical fiber tracts. In fact, at the time of DBS surgery, dopaminergic denervation and drug-induced sensitization have already advanced due to long-term dopaminergic, pulsatile treatment with consequently more severe neuropsychiatric symptoms [37]. The focal effect of neurostimulation in contrast to the global systemic effect of L-dopa contributes to the imbalance of motor and neuropsychiatric symptom control [36]. Therefore, within the first few postoperative months, PD patients are at risk of neuropsychiatric adverse effects of STN-DBS, with consequently higher, but transient, CB [38]. In contrast, in a long-term follow-up of 3–10 years of patients with STN-DBS, neuropsychiatric symptoms such as impulse control disorders and dopaminergic addiction were significantly reduced except for apathy and depression in 25% of PD patients after surgery [36,39]. Apathy is directly associated with decreased patient satisfaction [40] and might be related to delayed, postoperative dopamine agonist withdrawal [41] and, as a consequence, can lead to caregiver overload [42]. Importantly, suicide behavior can occur after STN-DBS in the first 3 years, with a higher risk of patients with psychotic symptoms and depression [9]. In conclusion, careful assessment of neuropsychiatric symptoms is recommended as they represent one of the main reasons for hospitalization of PD patients postoperatively [43]. Notably, neuropsychiatric symptoms such as depression, compulsivity and impulsivity increase post-DBS caregiver burden.

### 3.2. Caregiver Burden Outcome after STN-DBS Implantation of PD Dependants

**Figure 2:**
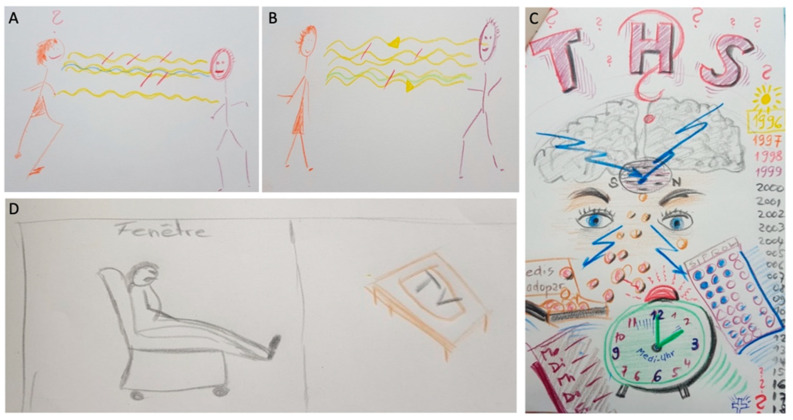
Individual perceptions of DBS effects by caregivers as published by [47]. A and B by Caregiver 1: preoperative (**A**) and postoperative (**B**) caregiver’s perception of her husband’s facial expression and improved communication after DBS. (**C**) Caregiver 2: caregiver’s perception of daily, chaotic, fast-paced timetables with appointments or tablet intake on time but hopeful eyes for better symptom control with DBS. (**D**) Caregiver 3: caregiver’s perception of postoperative fatigue and apathy of her husband.

There are unexpected, heterogeneous results of CB outcome after implantation of STN-DBS in PD. Despite improvement in motor function and higher social functioning of PD patients postoperatively, CB was variable and did not change in all caregivers 6 months after STN-DBS [38,44,45,46]. In a qualitative study with narrative semi-structured interviews and self-made drawings of PD patients and caregivers, heterogeneous reports were obtained [47]. Many patients and caregivers perceived the DBS “as the beginning of a new life”, “rebirth with clouds” and “renewal”, with improved “communication” and “better participation in everyday routines”. However, it was also conceived that “DBS is not perfect”, and, postoperatively, life still means “living with a sick person” [47] (Figure 2)

The divergent effects of STN-DBS on CB are already obvious in early reports of potential behavioral modifications in PD patients and its impact on familiar relations. In a cohort of 15 PD patients 6 months after DBS, 70% were reported to develop a euphoric mood due to symptom reduction but also fear of potential DBS failure and to return back to the preoperative level. This was associated with hostile behavior of the caregivers in the sense of worries to lose the newly acquired social and relative role again [48]. In another cohort of 29 PD patients, several difficulties of social adjustment despite marked motor improvement were observed 18–24 months after STN-DBS, resulting in disturbances of marital relationships [49]. Although DBS therapy was shown to improve QOL of PD patients without increasing CB in a group of 275 DBS patients [31], QOL and CB were rated worse by a considerable portion of caregivers [45]. An unfavorable caregiver satisfaction was even described, with approximately 50% of caregivers being disappointed with DBS outcomes [46]. This finding is astonishing since one would expect CB of relatives to decrease along with QOL and motor improvement of PD patients.

There might be several factors contributing to this incongruent development of QOL of patients and caregivers postoperatively.

**Figure 3:**
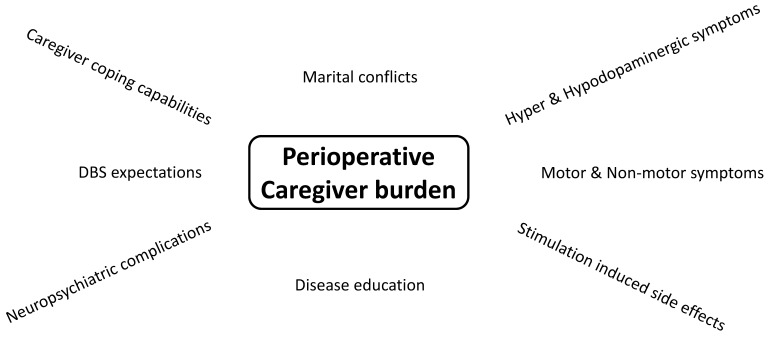
Pre-and postoperative mediators of caregiver burden of partners of PD patients with DBS.

1. The preexisting neuropsychiatric and medical condition of the caregivers themselves might play a role in the development of postoperative CB. In a prospective, longitudinal study of 25 patients and caregivers with follow-ups at 3 and 12 months, predictive factors for postoperative CB in a logistic regression model were assessed for both caregivers and PD patients [50]. Interestingly, at the 3-month follow-up, the caregivers were more indecisive about their own well-being but at the 1-year follow-up determined. There were caregivers with improved postoperative CB, often reporting “more freedom and better QOL” and that “PD patients showed less unpleasant side effects of medication”. Caregivers with worsening of postoperative CB reported “more conflicts between patients and caregivers”, “more anxiety concerning welfare of the patient” and “more sadness, stress and less freedom”. There were predicting risk factors of caregivers’ characteristics at baseline for increased postoperative CB, such as older age, greater depression, enhanced anxiety and lower quality of life of the caregivers [50]. This finding was interpreted as decreased ability of coping strategies or capabilities in those caregivers to adapt to the new postoperative situation [50]. Higher age of the caregiver is one important mediator of postoperative CB and caregivers’ satisfaction with the DBS operation. [46]. One has to remember that the caregiver grows older along with the PD patient and might also suffer from illnesses. The older the caregiver, the more exhausting the caregiving. Importantly, the preoperative BDI score is the predictor of postoperative caregiver depression one year after DBS surgery [46]. Thus is why the well-being of the caregiver should also be regularly addressed both pre- and postoperatively.
2. The postoperative extent of neuropsychiatric symptoms within PD patients significantly influences the CB of their relatives, as described above. Interestingly, CB was not associated with the extent of motor symptom improvement [44] but the patient’s degree of apathy and depression [2,50]. In 64 PD patients and caregivers, postoperative caregiver burden, as measured by the Zarit Burden Interview (ZBI), was significantly related to PD patients’ Beck Depression Inventory (BDI) score, caregiver-rated attentional impulsiveness of PD patients, impaired PD set-shifting, prepotent PD inhibition, patients’ hypersexuality and dopaminergic medication dose [2]. Higher postoperative CB, indexed by ZBI, was correlated with lower relationship quality [2]. In a second step, it was then assessed whether the new onset of stimulation-induced neuropsychiatric side effects in the early perioperative period of 6 weeks was a predictive factor for increased postoperative CB. There was a significant difference between caregivers of PD patients with and without DBS-induced neuropsychiatric side effects. CB decreased after 6 weeks compared to baseline in caregivers of PD patients without DBS-induced neuropsychiatric side effects but increased in a considerable portion of caregivers of PD patients with DBS side effects [2].
3. Postoperative marital conflicts due to changes in the relationship affect CB. DBS surgery profoundly changes caregiver responsibilities and disease-related symptoms due to the sudden relief of disability. Following STN-DBS, social maladjustment as a result of a dramatic improvement in clinical status and identity challenges can occur as part of the “burden of normality” syndrome [35]. Interestingly, partners of patients with device-aided therapies report more often changes in relationship satisfaction than patients and show more attachment-related avoidance [51]. Surprisingly, 65% of PD patients experience a conjugal crisis within 2 years of undergoing DBS [26]. Marital conflicts occurred in 17/24 couples, with three couples being divorced postoperatively and 33% of spouses becoming depressed within the 2-year follow-up [49]. Caregivers rate the change in their partnership 1 year post-DBS surgery as negative, with three main sources for marital disturbances: (1) neuropsychiatric changes, as described above with the new onset or ongoing neuropsychiatric symptoms in PD patients; (2) communication problems, e.g., caregivers feel uncertain of how to speak of residual PD symptomatology while the spouse feels healthy postoperatively; (3) caregivers’ overload of responsibilities for the partner and uncertainty about how the load of caregiving develops in the future [52]. About 54.5% of caregivers of PD patients with and without DBS suffer from caregiver overload [42]. During the time that partners spend together because of caring responsibilities, they feel emotionally more distanced [53]. These factors might contribute to an increase in marital conflicts [46]. Marital conflicts are further due to additional changes of social roles within the partnership postoperatively. On the one hand, there is a loss of the caregivers’ attention to the disease condition and the re-emerged responsibility in everyday duties for the PD patients, that PD patients might complain about [48]. Additionally, it was hypothesized that marital conflicts are due to role conflicts according to adaption to the new situation. On the other hand, PD patients rejected their spouse because they regained autonomy by DBS-induced motor improvement, but the spouse could not give up the caregiver role, overprotecting the patient and trying to maintain the patients’ dependency. Besides, patients were also rejected by their spouses, who expected them to resume a normal life with all their duties and social matters after the operation, whereas the patients still felt not able to do this [49]. However, some PD patients with good clinical outcomes also experience a restoration of the “old premorbid self” confirmed by their caregivers. Consequently, in those specific couples with satisfying DBS outcome, CB is lowered, and the relationship flourishes with greater socialization [26]
4. DBS is a symptomatic, but not disease-modifying therapy; thus, in the long-term, disease progression with re-emergence of motor symptoms, onset of cognitive impairment and loss of autonomy of PD patients might result in the reoccurrence of increased CB [54]. This might contribute to the observation that CB increases in caregivers of some PD patients within the first 2 years after STN-DBS [55]. However, in another 2-year follow-up of younger (<65 years) and elderly (>65 years) STN-DBS patients, CB decreased in relatives of both patient groups, even in caregivers of elderly PD patients [56]. Observations of long-term results of chronically stimulated patients are of particular interest in the growing numbers of operated and aging PD patients. In terms of PD symptomatology, there is increasing knowledge of long-term effects [57]. There is evidence that subthalamic nucleus DBS improves motor function for up to 10 years, although the magnitude of improvement, particularly of levodopa-resistant symptoms, tends to decline over time. Dyskinesia, motor fluctuations and activities of daily living in off-periods remain improved at 5 years, but quality-of-life scores usually decrease [57]. Nonetheless, in an observational period of about 14 years after STN-DBS surgery, the risk for recurrent falls and psychotic symptoms is lower in PD patients with STN-DBS compared to patients without DBS surgery [58]. Overall, patients’ satisfaction with DBS remains high at long-term follow-up of more than 6 years [7,59]. In a small cohort of late-stage PD patients (Hoehn and Yahr stage ≥4) with marked motor and cognitive impairment treated with STN-DBS for >14 years, patients still benefit partly from stimulation [60]. Interestingly, in this specific patient cohort, caregivers had mild to moderate stable caregiver burden. When the medical decision was made to discontinue DBS stimulation due to the clinical impression of poor stimulation response in this advanced disease stage, PD patients’ global motor state and dysphagia declined with delayed onset, while caregivers’ QOL and CB worsened after DBS discontinuation slightly, but without statistical significance [60]. The observation of mild-moderate CB in this small cohort of very advanced PD patients with a long disease duration of up to 43 years is astonishing and could be due to acceptance of the CB and habituation to the caregiving situation. Still, long-term observations of CB are scarce and need to be obtained in larger cohorts of long-term caregivers (Figure 3).

## 4. Caregiver Burden in PD Patients with Globus Pallidus Internus Stimulation

The globus pallidus internus (Gpi) is an alternative DBS target for PD patients besides STN implantation as the Gpi is supposed to have a lower risk of dysarthria, neuropsychiatric complications and impaired cognition [61]. The discussion of the favorable target in PD is still controversial [61]. There is also scarce information on caregiver burden in Gpi patients [31,62]. In a cohort with 275 DBS patients, including PD patients implanted both in STN and Gpi, caregiver burden measured by the Multidimensional Caregiver Strain Index (MCSI) was associated with PD age at surgery and interval since surgery [31]. Overall, QOL increased in this specific DBS cohort, whereas CB did not [31]. In a parallel two-arm clinical trial with 44 DBS patients, of which 81% were implanted in the Gpi, postoperative home health management with standard of care (SOC) postoperative management was compared [62]. In this cohort, caregiver burden measured by MCSI was reduced in both groups 6 months after surgery [62]. Interestingly, the MCSI was more reduced in the SOC than in the home health group, although the result did not reach significance [62]. In a large multicenter study on 1835 PD patients with a ≥10-year disease duration, 411 patients with DBS were included without further distinction of DBS targets. QOL worsened parallel to increased CB, which can mostly be explained by more severe symptom severity in advanced PD stages [63]. In summary, it appears that CB of partners of PD patients with STN and Gpi is mostly reduced shortly after DBS implantation but can increase over time with disease progression and reduced QOL. Further research in larger cohorts is needed on CB in caregivers of PD patients with Gpi stimulation.

## 5. What Is Next? Future Caregiving Challenges

DBS research is further advancing and new promising technologies are in the pipeline. Closed-loop systems with beta oscillations as internal biomarkers for independent, self-regulated adaption of stimulation are one example of current technological developments, which might improve PD patient outcome, reduce neuropsychiatric side effects and thus could decrease CB [64,65]. Still, these new systems might lead to increased DBS programming burden which might increase caregivers’ burden due to management of medical appointments and transportation. Another promising field is telemedicine with remote access for DBS programming. An obstacle in the treatment of PD patients is the fact that the optimization of DBS parameters is often performed only by movement disorders specialists at specific, far-away university hospitals, resulting in difficult transport and care coordination [66]. Telemedicine has already proven to reduce CB due to more flexible patient treatment and reduction of transportation issues to outpatient clinics [67]. Telemedicine comprises teleconsultation, telemonitoring and teletreatment [68]. Telehealth with teleprogramming of DBS has become particularly important during the COVID-19 pandemic with overall satisfactory patient experience [69,70]. Providing caregiver support via an online telecare system with constant monitoring of PD symptoms might improve QOL of DBS patients in the future [71]. Still, heterogeneous results of virtual house calls or telemedicine were observed in a general group of PD patients [72], thus the benefit of telemedicine and DBS teleprogramming for PD patients and caregivers needs to be proven in larger, multicentric studies. Another rising field represents trained PD nurses as part of an integrated care concept to reduce CB [73]. To face the rising demand for intensified caregiving, new models of care are implemented to empower patients and caregivers and personalize care management [74]. These efforts are reasonable to prevent institutionalization since a higher caregiver burden has been associated with higher risk of rehospitalization [75]. Interestingly, home health postoperative management of DBS patients revealed a decrease in clinic visits but without change in CB [62], indicating the need to optimize an integrated, multimodal concept in the postoperative management of DBS patients.

## 6. Conclusions

Informal caregivers play an important role in the daily care of PD patients before and after DBS surgery. Caregiver burden does not improve in all caregivers after STN-DBS and GPI-DBS in contrast to dramatic improvement of motor and non-motor symptoms and quality of life in PD patients. Relevant factors for postoperative CB are caregiver coping capabilities, postoperative onset of neuropsychiatric symptoms of PD patients, marital conflicts and the awareness of the symptomatic nature of DBS therapy. But, There is still a lack of information on long-term caregiver burden and predictors after STN-DBS.

Potential tools to reduce postoperative CB represent “preparedness of the caregivers”, which could be protective against postoperative caregivers distress [76]. Caregivers should be informed about the specific postoperative aspect of STN-DBS and the potential effect on their own QoL. They should be more intensively integrated into the pre- and postoperative processes. Another option to reduce CB of STN-DBS patients could be cognitive behavioral therapy for caregivers [77], which could substantially modify CB prior to and after STN-DBS surgery. Additionally, self-management programs to retain social participation can help the caregiver to maintain well-being during the course of the disease [78,79].

To sum it up, there are heterogeneous results on CB changes after STN-DBS, but there are therapeutic approaches to reduce CB in the future. Increased awareness of the special perioperative demands of PD patients with STN-DBS and caregivers is fundamental for an optimized future approach.

## Figures and Tables

**Table 1:** Overview of included studies on caregiver burden in DBS PD patients. Studies were identified in PubMed with the search terms “Caregiver Burden” and “Deep Brain Stimulation” or “DBS”. C: Control group; DBS, Deep brain stimulation; E: Experimental group; MSCI; Multidimensional Caregiver Strain Index; PD, Parkinson’s disease; QOL, Quality of life; SD, Standard deviation; y, years; ZBI, Zarit burden Interview.

Study	Design	PD Age, y ± SD	PD Sample Size	PD Duration, y ± SD	PD Sex (M/F)	Numbers of Caregivers Enrolled	Domain of Interest/Outcome Parameters
Duffley et al., 2021	Parallel, randomized controlled trial	E: 65.0 ± 10.9 SOC: 64.1 ± 10.0	E: 23 SOC: 19	E: 12.0 ± 5.9 SOC: 11.5 ± 7.2	E: 13/10 SOC: 11/8	E: 22 SOC: 14	Home health management of PD DBS, MSCI
Jackowiak et al., 2020	Retrospective	63.3 ± 8.1	35	10.6 ± 5.2	28/7	35	2-year follow-up after STN-DBS surgery, Caregiver Burden Inventory
Mosley et al., 2021	Prospective clinical trial	-	-	-	-	10	Cognitive behavioral therapy for caregivers of PD patients with STN-DBS, ZBI, Parkinson’s Disease Questionnaire-Carer
Macchi et al., 2019	Secondary analysis of randomized controlled trial	70.6 ± 8.1	170 (20 with DBS)	9.5 ± 6.5	70/119	170 (20 with PD Patients with DBS)	Physical/sexual aggression, ZBI Caregiver burden, Caregiver-perceived QOL, caregiver anxiety
Vats et al., 2019	Retrospective	<65 y: 51.92 ± 8.2 >65>: 68.75 ± 3.05	<65 y: 20 >65>: 20	- -	<65 y: 13/7 >65 y: 7/5	<65 y: 20 >65>: 20	2-year follow-up after STN-DBS surgery, ZBI
Mosley et al., 2018	Prospective clinical trial	62.2 ± 9.5	64	9.0 ± 5.2	48/16	64	26-week follow-up after STN-DBS surgery, ZBI, Relationship Quality Inventory, Barratt Impulsiveness Scale, Caregiver-rated Empathy Quotient
Witt et al., 2017	Scientific contribution	-	21	-	14/7	21	Case study with semi-structured qualitative interviews, 1-year follow-up after DBS surgery, changes in partnership (psychological changes, communication problems, overload)
Crespo-Burillo et al., 2018	Cross-sectional observational study	66.2 ± 7.1	11	21.5 ± 17	7/4	11	Zarit Caregiver Burden Interview, Hospital Anxiety and Depression Scale
Soileau et al., 2014	Retrospective	66.5 ± 7.2	12	10.6 ± 4.7	9/3	12	6-month follow-up after STN-DBS surgery, Caregiver Burden Inventory
Oyama et al., 2014	Cross-sectional retrospective study	62.6 ± 8.8	275	15.0 ± 6.3	-/-	275	MSCI
Hassan et al., 2012	Multicenter study	67.8 ± 9.5	1835 (411 with DBS)	15.1 ± 5.3	1141/693	1617 (88.1% of PD patients with regular caregiver)	PD patients with disease duration ≥ 10 years, MSCI

## Data Availability

No report of data.

## Abbreviations

ADL, Activities of daily living; APPLIQue, Alzheimer’s Patient Partners Life Impact Questionnaire (APPLIQue) BDI, Beck Depression Inventory, CB, Caregiver burden; DBS, Deep brain stimulation; L-dopa, levodopa; MCSI, Multidimensional Caregiver Strain Index; MDS-UPDRS, MDS-Unified Parkinson’s Disease Rating Scale; PD, Parkinson’s disease; QOL, Quality of life; SOC, Standard of Care; STN, Nucleus subthalamicus; ZBI, Zarit Burden Interview.
